# Tenosynovitis caused by *Trichosporon asahii* in an immunocompetent patient

**DOI:** 10.31744/einstein_journal/2025RC1095

**Published:** 2025-04-07

**Authors:** Lais Lopes Pires, Mayara Gabriele Toledo, Hugo Rodrigues Alves, Isadora Cambruzzi, Guilherme Brezenski Rodrigues, Andrea D'avila Freitas, Natalia Chilinque Zambao da Silva

**Affiliations:** 1 Complexo Hospital de Niterói Niterói RJ Brazil Complexo Hospital de Niterói, Niterói, RJ, Brazil.; 2 Universidade Federal Fluminense Hospital Universitário Antônio Pedro Niterói RJ Brazil Hospital Universitário Antônio Pedro, Universidade Federal Fluminense, Niterói, RJ, Brazil.; 3 Instituto Nacional de Infectologia Fundação Oswaldo Cruz Rio de Janeiro RJ Brazil Instituto Nacional de Infectologia, Fundação Oswaldo Cruz, Rio de Janeiro, RJ, Brazil.

**Keywords:** Mycoses, Yeasts, Tenosynovitis, Trichosporon, Trichosporon asahii, Voriconazole

## Abstract

Fungal infections have become a public health problem owingdute to their progressive increase in recent decades and high morbidity rates. Fungal bone and joint infections result from direct inoculation, contiguous infection spread, or hematogenous seeding of organisms. *Trichosporon spp*. are yeast-like basidiomycetes, with *Trichosporon asahii* being the most common pathogenic species. This article describes a rare case of tenosynovitis caused by *Trichosporon asahii* in an immunocompetent patient. Treatment with voriconazole resulted in an excellent clinical response.

## INTRODUCTION

*Trichosporon* genus includes various widely distributed basidiomycetous yeasts that are often found in natural environments and animals.^(1)^ Additionally, they are part of the human microbiota in the skin and gastrointestinal and respiratory systems, although specific species are potential pathogens.^(2)^

*Trichosporon* includes six species of medicinal interest-*T. ovoid, T. inkin, T. asahii, T. asteroid, T. mucoides, and T. cutaneum (or T. beigelli). T. asahii* and *T. mucoides* are associated with deep infections, whereas *T. asteroides, T. ovoids*, and *T. cutaneum* are responsible for white piedra and other superficial infections. *T. inkin* causes both superficial and deep infections. These pathogens grow on Sabouraud Dextrose agar; however, their identification remains unclear.^(3-5)^

*Trichosporon spp.* Infections are divided into superficial and invasive, with various clinical manifestations. Superficial infections, such as piedra branca and onychomycosis occur in immunocompetent individuals, whereas invasive trichosporonosis occurs in patients with compromised immunity.^(3)^ The first case of disseminated *Trichosporon spp.* was reported in 1970 in a patient with leukemia. Since then, cases have been increasingly documented in the literature.^(6)^

Predisposing factors for *Trichosporon spp.* infections include immunosuppression, invasive procedures, prolonged hospitalization, chronic diseases, organ transplantation, acquired immunodeficiency syndrome, extensive burns, and use of chemotherapy and antibiotics.^(4)^

*T. asahii*, formerly known as *T. beigelii*, is traditionally associated with opportunistic infections in patients with compromised immunity, causing disseminated infections.^(4)^

This study aimed to report a rare case of an invasive *T. asahii* infection in an immunocompetent patient, a condition rarely documented in the literature.

## CASE REPORT

**Figure 1:**
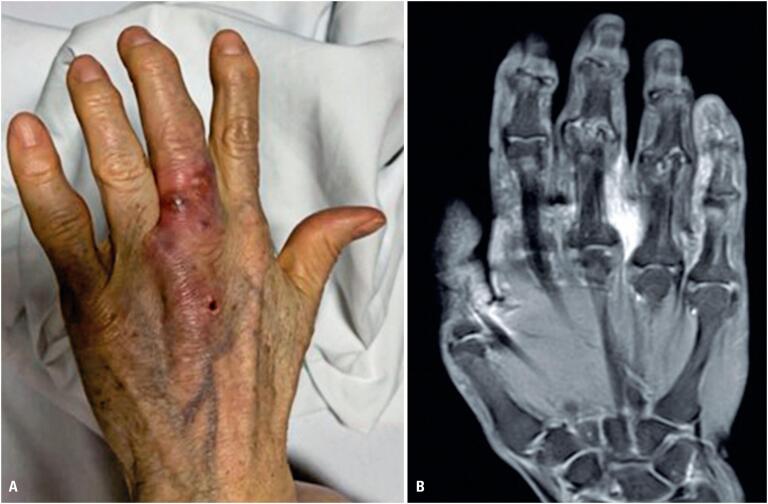
(A) Left hand injury with redness, edema, and nodulation in the third finger; (B) Magnetic resonance imaging of the left hand revealed a partially defined spindle-shaped tissue between the 3^rd^ and 4^th^ metacarpals, adjacent to the extensor tendons, involving the medial aspect of the proximal phalanx of the third finger, with adjacent liquid/edematous tissue infiltration

A 67-year-old female Brazilian patient with no history of trauma or surgery at the affected site presented to the emergency room in May 2022 with pain, redness, and nodulation of the third finger of her left hand. Her medical history included hypothyroidism, fibromyalgia, anxiety disorder, and rheumatoid arthritis, for which she had been treated with leflunomide for five years. Magnetic resonance imaging of the left hand revealed a partially defined spindle-shaped tissue mass (4.2×1.2×9.5cm) centered in the subcutaneous tissue of the dorsal region of the hand between the 3^rd^ and 4^th^ metacarpals. The mass was adjacent to the extensor tendons, protruded into the interphalangeal region, and involved the medial aspect of the proximal phalanx of the third finger with adjacent liquid/edematous tissue infiltration (Figure 1). The patient was diagnosed with tenosynovitis.

The patient underwent orthopedic surgery on May 9, 2022. Intraoperatively, multiple abscesses and granulomas with the degeneration of the extensor apparatus of the middle finger were visualized macroscopically. Empirical therapy with vancomycin was administered postoperatively for six days until the cultures tested negative for common pathogens, after which it was discontinued. On May 14, 2022, a fungal culture revealed the presence of *T. asahii*. Intravenous voriconazole (200 mg every 12 h) was administered for five days, followed by oral treatment. The patient was discharged after a total treatment duration of 12 weeks.

This study was approved by the Research Ethics Committee of *Hospital Nove de Julho* (CAAE: 47504821.0.0000.5455; # 5.543.350).

## DISCUSSION

*T. asahii* is a non-*Candida* yeast that colonizes the skin, mucosa, and gastrointestinal, respiratory, and genitourinary systems.^(7,8)^ Infections caused by the fungus are severe, invasive, and occur primarily in patients with compromised immunity, with rare cases in immunocompetent children.^(7-9)^ The disease presents insidiously in various clinical forms, requiring a high degree of suspicion for diagnosis.^(9,10)^

An epidemiological study by Li et al. reported that numerous *T. asahii* infections have been documented in China; however, few have been reported in developed countries. In these countries, the suspicion of trichosporonosis may have been delayed because of clinical unfamiliarity with the infection.^(9,11)^ Additionally, the lack of data from various global regions was mentioned, emphasizing the need for further studies to explore the disease's pathogenesis, infectious mechanisms, and optimal antifungal treatments.^(11,12)^

Regarding the intraspecific variety of *T. asahii*, a multicenter study by Francisco et. al. identified five circulating genotypes in Brazilian patients: G1, G3, G4, G5, and G7, with G1 genotype as the most prevalent.^(10)^

There are a few cases of *Trichosporon* infections in the medical literature, and the first reported tenosynovitis caused by *Trichosporon* was published in 2009. Invasive trichosporonosis is a rare, but fatal, opportunistic infection. The mortality rate among infected patients varies widely based on the infection severity and site, although it can range from 50-80 % in certain cases.^(9,11)^ After *Candida*, *Trichosporon* species are the second most isolated pathogens in patients with cancer.^(1)^

Antibiotic therapy, invasive medical equipment, neutropenia, and hematological malignancies are recognized as the primary risk factors for trichosporonosis, in addition to other immunosuppressive conditions and minor risk factors.^(12)^ Patients with cancer and severe neutropenia are specifically vulnerable.^(7,8)^ Our patient was administered leflunomide, an immunosuppressive drug that inhibits dihydroorotate dehydrogenase-an enzyme essential for pyrimidine production. Pyrimidines are primary components of DNA synthesis and cell proliferation. By blocking this enzyme, leflunomide reduces immune cell proliferation, thereby increasing susceptibility to fungal infections.

Gram-positive organisms are well-known causes of tenosynovitis in immunocompetent adults.^(13)^ The most common isolate associated with hand infections is *Staphylococcus aureus*, which is also the primary agent in infections following clean surgical procedures. For implant-related infections, *Staphylococcus epidermidis* is the most frequent pathogen.^(13)^ There are few reported cases of *Trichosporon* infection in immunocompetent individuals.^(9)^

The diagnosis of *Trichosporon* infections is based on clinical presentation and confirmed by microscopy and culture. Samples can be collected from blood, sputum, and urine.^(9)^

The treatment for trichosporonosis is challenging.^(7,9)^ Literature indicates that the use of voriconazole, with or without other antifungals, is an effective treatment option. Echinocandins or amphotericin B are not recommended for treatment or prophylaxis because of their ineffectiveness.^(7,8,10)^ In cases with severe complications, surgery may be required in addition to antifungal therapy.^(9)^ In this case, there was significant clinical enhancement with the use of voriconazole, without the need for amphotericin B or surgery. Despite the recommended treatments, *Trichosporon* infection remains a potentially fatal condition.^(7,9)^

## CONCLUSION

Trichosporonosis is an emerging infection that often presents in an invasive form and is a cause of concern. The infection is considered to be more severe because of its inherent resistance to commonly used antifungal drugs. Early diagnosis and appropriate antifungal treatment are crucial for managing the infection and preventing complications. However, studies on *T. asahii* infections, specifically in developed countries, are lacking.
